# Expression of substance P, calcitonin gene-related peptide and vascular endothelial growth factor in human dental pulp under different clinical stimuli

**DOI:** 10.1186/s12903-021-01519-x

**Published:** 2021-03-23

**Authors:** Javier Caviedes-Bucheli, Luis Fernando Lopez-Moncayo, Hernan Dario Muñoz-Alvear, Jose Francisco Gomez-Sosa, Luis Eduardo Diaz-Barrera, Hernando Curtidor, Hugo Roberto Munoz

**Affiliations:** 1 Centro de Investigaciones Odontologicas, Pontificia Universidad Javeriana, Bogotá, Colombia; 2 Endodontics Department, Universidad Cooperativa de Colombia, Pasto, Colombia; 3 Postgraduate Endodontics Department, Universidad Central de Venezuela, Caracas, Venezuela; 4 Engineer School, Universidad de La Sabana, Chía, Colombia; 5 Universidad del Rosario, Bogotá, Colombia; 6 Endodontics Department, Universidad de San Carlos de Guatemala, Guatemala City, Guatemala

**Keywords:** SP, CGRP, VEGF, Occlusal trauma, Orthodontics force, Neurogenic inflammation

## Abstract

**Background:**

The aim of this study was to measure the dental pulp inflammatory response through neuropeptides (SP and CGRP) as a response to occlusal trauma, orthodontic movements and a combination of both, as well as the angiogenic defense mechanism through VEGF expression, which could be the initial step to mineralized tissue formation.

**Methods:**

Forty human dental pulp samples were collected from healthy first premolars with extraction indicated due to orthodontic reasons from a sample of 20 patients. Patients were divided into four groups with 10 premolars each (1 mandibular and 1 maxillary premolar from each patient): healthy pulp control group, occlusal trauma group, moderate orthodontic forces group; and occlusal trauma plus moderate orthodontic forces group. Stimuli were applied for 24 h before tooth extraction in all experimental groups. All samples were processed, and SP, CGRP, and VEGF were measured by radioimmunoassay. The Kruskal–Wallis test was performed to assess significant differences among groups and Mann–Whitney’s U post hoc pairwise comparisons were also performed.

**Results:**

The highest increase in SP, CGRP, and VEGF expressions was found in the occlusal trauma plus orthodontic forces group, followed by the moderate orthodontic forces, the occlusal trauma and the control groups, with statistically significant differences between all groups for each of the 3 peptides analyzed (Kruskal–Wallis *p* < 0.001). All possible pairwise post-hoc comparisons were also significant for each peptide analyzed (Mann–Whitney’s U *p* < 0.001).

**Conclusion:**

SP, CGRP, and VEGF expressions significantly increase in human dental pulps when stimulated by occlusal trauma combined with moderate orthodontic forces, as compared with these two stimuli applied independently.

- Name of the registry: Importance of Neurogenic Inflammation in the Angiogenic Response of the Dental Pulp as a Defensive Response.
- Trial registration number: NCT03804034.
- Date of registration: 01/15/2019 Retrospectively registered.
- URL of trial registry record: https://clinicaltrials.gov/ct2/show/NCT03804034?term=NCT03804034&draw=2&rank=1.

## Background

When stimulated, the dental pulp can physiologically change its vascular, neural, and cellular components in order to adapt to masticatory function or events such as occlusal trauma and orthodontic forces. These stimuli may trigger a neurogenic inflammation through neuropeptides, such as Substance P (SP) and Calcitonin gene-related peptide (CGRP) [1], which may initiate a defense response and angiogenesis to prevent functional changes that could lead to necrosis [2–4].

Each neuropeptide must bind to a specific receptor in order to exert a biological function: for SP, the NK1 receptor is found in mast cells, macrophages, mesenchymal cells, fibroblasts, endothelial cells, and odontoblast-like cells [1]. This SP/NK1 complex had vascular effects, such as blood flow regulation and vasodilation, as well as inflammatory effects, promoting inflammatory cells chemotaxis and collagen formation as a defense mechanism [5].

SP/NK1 complex stimulates angiogenesis using direct mechanisms that modulate the endothelial cells and fibroblasts action by activating growth factors such as vascular endothelial growth factor (VEGF) and stimulating cell migration and proliferation to form mineralized tissues as a defense mechanism [2, 5, 6]. This complex also uses indirect mechanisms, such as binding to granulocytes or macrophages, attracting cells with angiogenic potential [4, 7].

The CGRP exerts its biological activity by binding to CGRP receptors 1 or 2 (CGRPR1-2), with the former being the most sensitive protein to CGRP. CGRPR1 is a potent vasodilator that increases intrapulpal blood flow [8]. It is found on endothelial cells, mast cells, macrophages, lymphocytes, undifferentiated mesenchymal cells, fibroblasts, and odontoblast-like cells; and controls neurogenic inflammation and reparation processes combined with SP due to its angiogenic potential [1, 9].

Angiogenesis is mediated by several growth factors, including VEGF [10, 11] that is released in the pulp tissue as a response to a harmful stimulus to counteract emerging hypoxic areas by regulating oxygen and nutrient supply to cell populations that form new blood vessels [12, 13]. VEGF is also found in endothelial cells, mast cells, macrophages, lymphocytes, undifferentiated mesenchymal cells, fibroblasts, and odontoblast-like cells, controlling neurogenic inflammation and reparative processes combined with SP and CGRP due to its angiogenic potential [11, 14].

In patients undergoing orthodontic treatment, occlusal trauma and orthodontic forces are clinical situations that affect the human dental pulp, modifying its metabolism and causing vascular changes leading to hypoxia [4, 15]. The dental pulp response depends on the intensity, magnitude and duration of the stimulus. If the stimulus becomes constant, the resulting hypoxia will inevitably induce a defense response from the dental pulp via neurogenic inflammation and angiogenic mechanisms that could induce the formation of mineralized tissues [11, 13, 15–17]. The present research is intended to verify the inflammatory process provoked in dental pulp by occlusal trauma and orthodontic forces through neuropeptide expression and confirmation of the angiogenic defense mechanism through VEGF expression.

Thus, the purpose of this study is to measure SP, CGRP, and VEGF expression in human dental pulp under occlusal trauma or moderate orthodontic forces, as well as both stimuli combined.

## Methods

This experimental study was conducted following the recommendations of the Colombian Ministry of Health regarding ethical issues of scientific research involving human tissues. It was approved by the Bioethics Committee of the Faculty of Dentistry at the Universidad Cooperativa de Colombia in Pasto (CBE07). Written informed consent was obtained from all patients participating in this study. This study was registered in clinicaltrials.gov under the title: “Importance of Neurogenic Inflammation in the Angiogenic Response of the Dental Pulp as a Defensive Response”, trial registration number NCT03804034, dated 01/15/2019, URL: https://clinicaltrials.gov/ct2/show/NCT03804034?term=NCT03804034&draw=2&rank=1.

A convenient sample of 20 healthy patients, 8 men and 12 women, aged between 18 and 30 years was recruited. Human dental pulps were obtained from 20 maxillary and 20 mandibular first premolars with extraction indication due to orthodontic reasons. Patients taking medications for pain, smokers, and pregnant women were excluded. All the teeth included in this study had a normal masticatory function with a radiographically complete root development (clinically confirmed after extraction), without caries, restorations, periodontal disorders, or parafunctional habits.

**Fig. 1:**
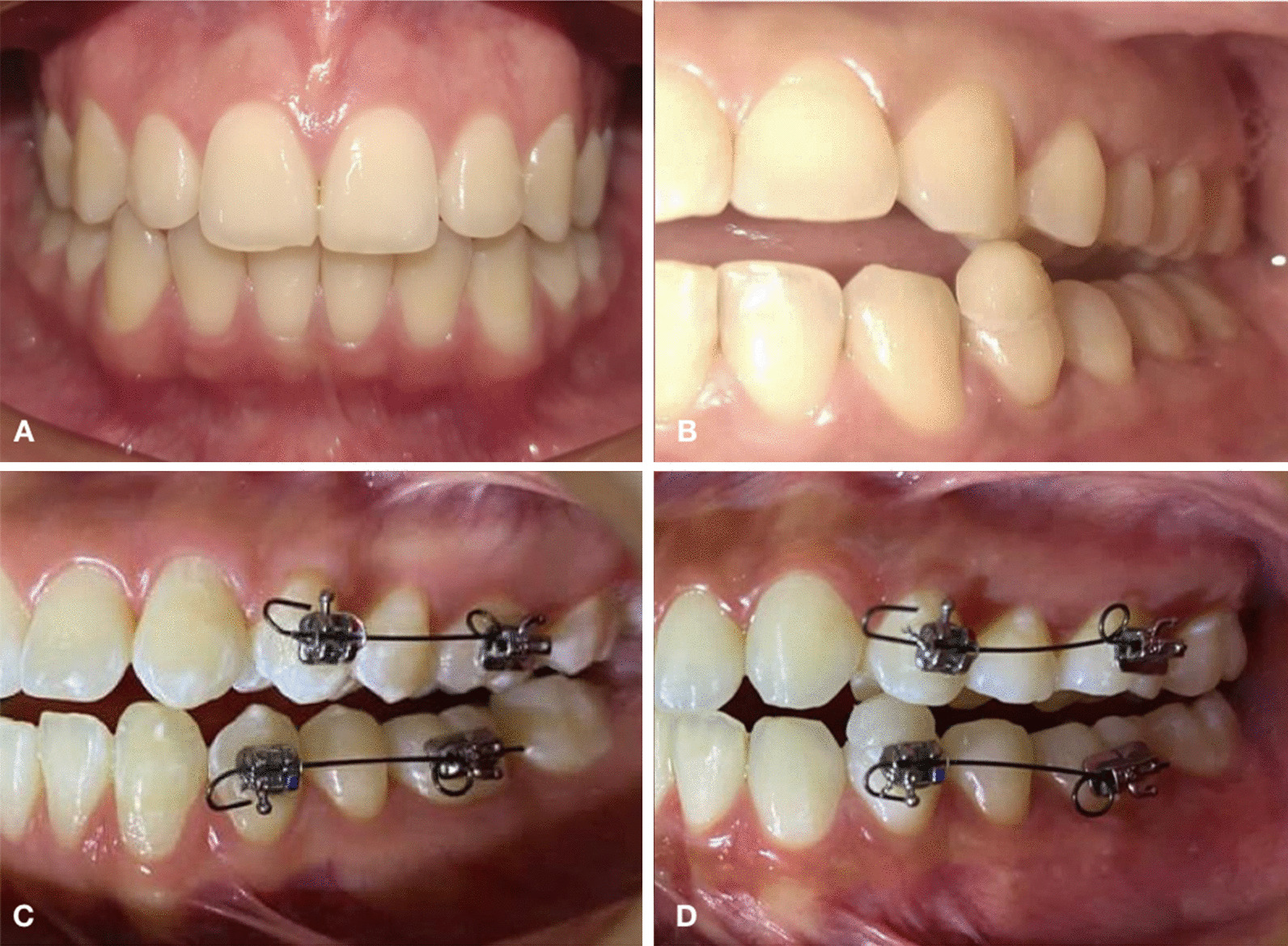
Clinical images of the different stimuli applied to the patients in each group. **a** Control Group (without occlusal trauma nor orthodontic force); **b** occlusal trauma group; **c** moderate orthodontic force group; **d** occlusal trauma plus moderate orthodontic force group

Patients were randomly assigned (Random.org App, Randomness and Integrity Services Ltd, Ireland) to each group for a total of 5 patients (10 premolars, 1 maxillary and 1 mandibular premolar from the same side of each patient) per group: (a) control group (maxillary and mandibular healthy premolars without any stimuli) (Fig. 1a); (b) occlusal trauma (1 maxillary and 1 mandibular premolars that were in contact under occlusal trauma from each patient); (c) orthodontic force (1 maxillary and 1 mandibular premolars with orthodontic forces applied for 24 h); (d) occlusal trauma and orthodontic force (1 maxillary and 1 mandibular premolars that were in contact under occlusal trauma and orthodontic force). Premolars included in this study were from the side where it was a greater surface contact between first maxillary and mandibular premolars. Contralateral teeth were also extracted for orthodontic reasons on the same appointment but were not used in the study.

In the occlusal trauma group (Fig. 1b), an occlusal interference was placed on the mandibular premolars. An articulating paper was used to mark the contact area between the maxillary and mandibular premolars indicated for extraction, following the study model reported by Caviedes Bucheli et al. [18]. Mandibular premolar was acid-etched with 37% phosphoric acid (SuperEtch; SDI Ltd, Australia) for 15 s. After washing and drying, a bonding agent (STAE; SDI Ltd) was applied and light-cured for 15 s, and finally a 2 mm resin block (Filtek Z350; 3 M Espe, Germany) was placed and light-cured (40 s) over the area where the maxillary premolar occluded. An articulating paper was used to verify that only the premolars indicated for extraction had contact during normal occlusion as well as in lateral movements. Patients were given chewing gums and were instructed to repeat 20 masticatory cycles for 30 s, followed by a 30 s rest interval, and repeat the sequence for 30 min. This chewing cycle was repeated three times every 8 h for the first 24 h and it was confirmed by each patient in a format with the established schedules.

For the moderate orthodontic force group (Fig. 1c), the occlusal surface of the first mandibular molar was raised using a block of resin (Filtek Z350; 3M Espe, Germany) until the premolars were out of occlusion before applying an orthodontic force, following the study model reported by Caviedes-Bucheli et al. [19]. Convertible standard buccal tubes (Orthorganizer, USA) were bonded on the buccal surface of the first maxillary and mandibular molar with resin (Light Bond; Reliance Orthodontic Products Inc, USA). A McLaughlin, Bennett, and Trevisi (MBT) 0.022 in slot bracket (Ref. 702-393 MC, Orthoorganizer) was bonded over the buccal surface of the maxillary and mandibular first premolars. A 0.017 × 0.025 in titanium molybdenum alloy (TMA) wire cantilever was inserted into each first molar tube and the wire was bent occlusally to form a helix with an activation angle of 45°. The cantilever was cinched to the distal end of the tube, and the orthodontic force was measured with a digital orthodontic dynamometer to approximately 56 g exerting an extrusive force on the premolar for 24 h before being extracted.

In the occlusal trauma and moderate orthodontic force group (Fig. 1d), occlusal interferences combined with orthodontic force were performed as previously described; this force was applied to the teeth for 24 h before being extracted, without placing the resin bumpers on the first molars.

### Sample collection

All the teeth extracted in this study were anesthetized with 1.8 ml of 4% prilocaine without a vasoconstrictor (Pricanest, Ropsohn Therapeutics Ltd. Colombia) by infiltrative injection for maxillary premolars and inferior alveolar nerve block injection for mandibular premolars. The teeth were extracted with conventional methods 5 min after administering the anesthetics. Immediately after the extraction, the teeth were sectioned using a Zekrya bur (Dentsply, USA) in a high-speed handpiece irrigated with saline solution. The pulp tissue was obtained using a sterile endodontic excavator, placed on an Eppendorf tube, snap frozen in liquid nitrogen, and kept at − 70 °C until the radioimmunoassay test was performed.

### Radioimmunoassay (RIA)

Tissue samples were defrosted without thermal shock, dried on a filter, and weighed on an analytic balance. Peptides were extracted by adding 150 μL of 0.5 M acetic acid and double boiled in a thermostat bath for 30 min.

SP expressions (Phoenix Peptide Pharmaceutical RK-061-05, USA) and CGRP (Phoenix Peptide Pharmaceutical RK-015-02, USA) were determined by competitive binding assays using the corresponding kits for each substance following previously published protocols [18–20]. The VEGF expression was equally determined by competition binding assays using a human, recombinant VEGF165 (G143AB; Genentech, Inc., USA), primary antibody, polyclonal rabbit antiserum to VEGF165 (27906-17, Genentech, Inc., USA), and human recombinant ^125^I-VEGF tracer (NEX328, NEN Life Science Products, Inc. USA). Each sample solution (50 μL) was incubated in tubes at 18 °C for 20 h with 100 μL of primary antibody, and 100 μL of different peptide concentrations (SP: 7.42 × 10^−3^ − 0.89 pmol/mL; CGRP: 2.64 × 10^−3^ − 0.32 pmol/mL; VEGF: 2.5 × 10^−4^ − 3.2 × 10^−2^ pmol/mL) was added with 50 μL of radiolabeled factors and incubated for 24 h. Bound fractions were precipitated with secondary antibody (100 μL), 100 μL of fetal bovine serum, and 500 μL of RIA buffer containing 1% prolyethylene glycol 8000. After 2 h incubation at 20 °C, the tubes were centrifuged at 3000 rpm for 45 min at 4 °C. The supernatants were decanted, and the pellet radioactivity was read in a gamma counter. The peptide concentration was determined according to calibration curves of the radioactivity in each mixture. A blinded operator processed and analyzed the pulp tissue samples, which were coded so the blinded operator could not know what stimulus was applied on the tissue being analyzed.

### Statistical analysis

Results are presented as SP, CGRP, and VEGF expressions in pmol/mg of pulp tissue. Means and standard deviations, as well as minimum and maximum values for each neuropeptide and VEGF, were calculated. Due to the small sample size, a Kruskal–Wallis test was used to assess statistically significant differences among groups, followed by Mann–Whitney’s U post-hoc tests with correction of statistical significance based on the number of comparisons (Significance level: 0.05/6).

## Results

**Fig. 2:**
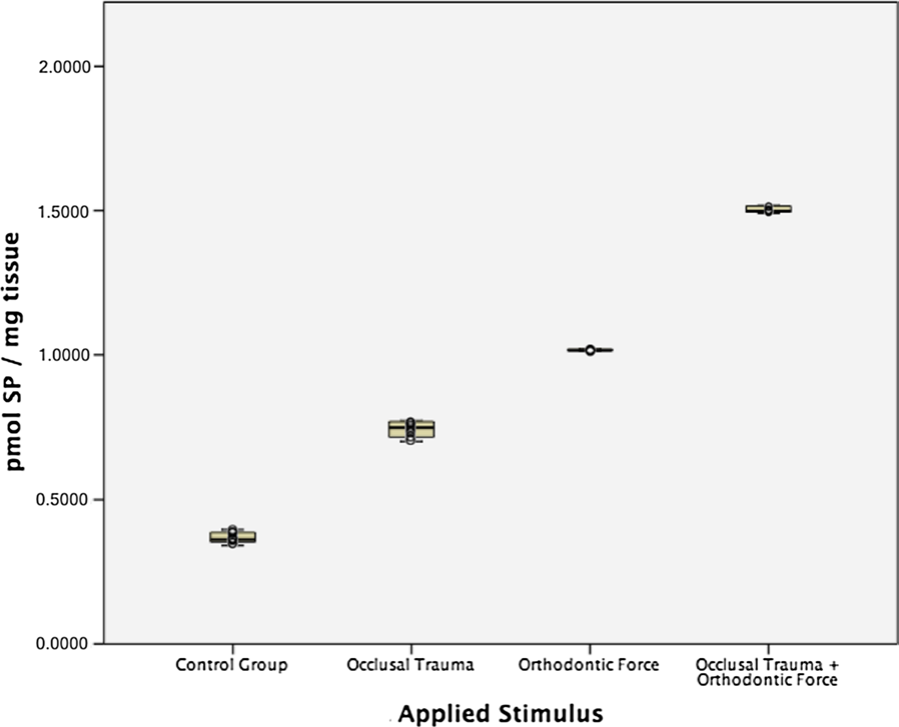
Substance P (SP) expression (pmol/mg) in human dental pulp from teeth under different stimuli (Colored boxes: mean and 95% confidence interval, whiskers: minimum and maximum values, circle dots: individual sample values)

SP, CGRP, and VEGF expressions were found in all 40 analyzed pulp tissue samples. The mean SP expression in the control group was 0.3665 ± 0.0198 pmol/mg of pulp tissue. For the occlusal trauma group, the mean SP expression increased to 0.7432 ± 0.028 pmol/mg; in the orthodontic force group, the mean expression was 1.0163 ± 0.0025 pmol/mg (Table 1). The highest SP expression was found in the occlusal trauma plus orthodontic force group, with a mean expression of 1.5021 ± 0.0104 pmol/mg (Fig. 2). Kruskal–Wallis test found statistically significant differences among groups (*p* < 0.001) and Mann–Whitney’s U test showed statistically significant differences in all possible pairwise comparisons (*p* < 0.001).

**Table 1:** SP expression in human dental pulps of teeth with occlusal trauma, orthodontic force and a combination of both stimuli

Applied Stimuli^b^	No. of teeth	Mean^a^	SD	95% confidence interval	
Healthy premolars control group	10	0.3665	0.0198	0.3524	0.3808
Occlusal Trauma	10	0.7432	0.0281	0.7231	0.7634
Orthodontic Force	10	1.0163	0.0025	1.0145	1.0181
Occlusal Trauma + Orthodontic Force	10	1.5021	0.0104	1.4947	1.5096

^a^Values are presented as SP concentration in pmol per mg of dental pulp

The Kruskal–Wallis test showed statistically significant differences among groups (*p* < 0.001)

^b^Mann-Whitney’s U post-hoc pairwise comparisons (with correction of significance) showed statistically significant differences in all possible pairwise comparisons (*p* < 0.001)

**Fig. 3:**
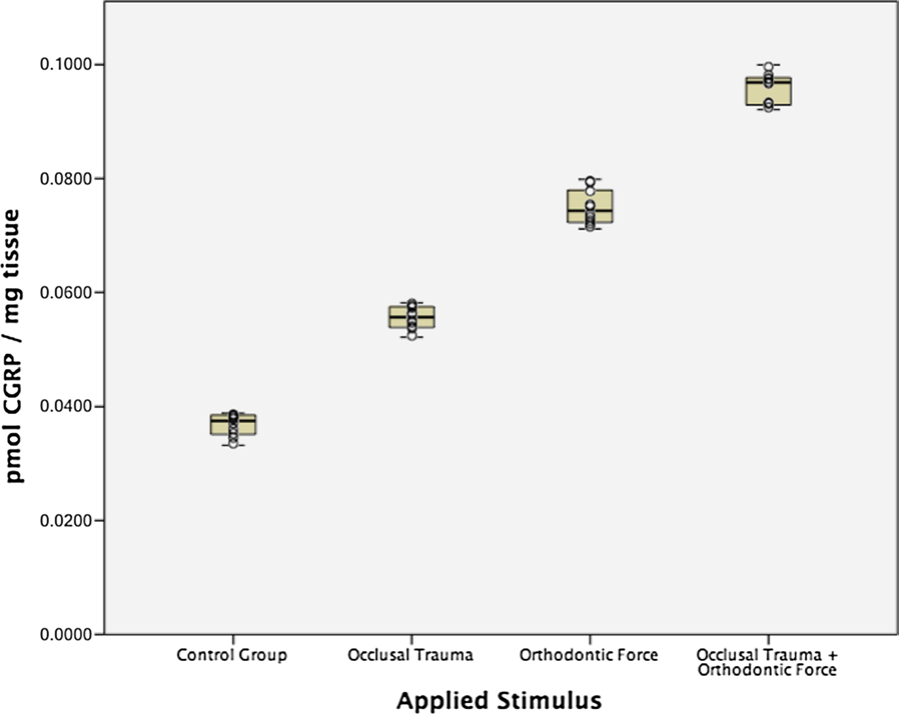
Calcitonin gene-related peptide (CGRP) expression (pmol/mg) in human dental pulp from teeth under different stimuli (Colored boxes: mean and 95% confidence interval, whiskers: minimum and maximum values, circle dots: individual sample values)

The mean CGRP expression in the control group was 0.0367 ± 0.0019 pmol/mg of pulp tissue. In the occlusal trauma group, the mean CGPR expression increased to 0.0555 ± 0.0020 pmol/mg; in the orthodontic force group, the mean expression was 0.0749 ± 0.0032 pmol/mg (Table 2). The highest CGRP expression was found in the occlusal trauma plus orthodontic force group, with a mean expression of 0.0958 ± 0.0028 pmol/mg (Fig. 3). Kruskal–Wallis test found statistically significant differences among groups (*p* < 0.001) and Mann–Whitney’s U test showed statistically significant differences in all possible pairwise comparisons (*p* < 0.001).

**Table 2:** CGRP expression in human dental pulps of teeth with occlusal trauma, orthodontic force and a combination of both stimuli

Applied Stimuli^b^	No. of teeth	Mean^a^	SD	95% confidence interval	
Healthy premolars control group	10	0.0367	0.0019	0.0354	0.0382
Occlusal Trauma	10	0.0555	0.0020	0.0541	0.0569
Orthodontic Force	10	0.0749	0.0032	0.0727	0.0773
Occlusal Trauma + Orthodontic Force	10	0.0958	0.0028	0.0938	0.0979

^a^Values are presented as CGRP concentration in pmol per mg of dental pulp

The Kruskal–Wallis test showed statistically significant differences among groups (*p* < 0.001)

^b^Mann-Whitney’s U post-hoc pairwise comparisons (with correction of significance) showed statistically significant differences in all possible pairwise comparisons (*p* < 0.001)

**Fig. 4:**
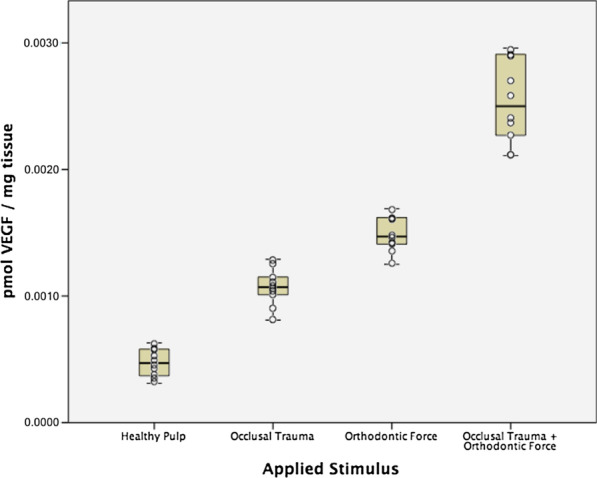
Vascular endothelial growth factor (VEGF) expression (pmol/mg) in human dental pulp from teeth under different stimuli (Colored boxes: mean and 95% confidence interval, whiskers: minimum and maximum values, circle dots: individual sample values)

Mean VEGF expression in the control group was 0.0004 ± 0.0001 pmol/mg of pulp tissue. For the occlusal trauma group, the mean VEGF expression increased to 0.0010 ± 0.0001 pmol/mg, followed by the moderate orthodontic force group with a mean expression of 0.0014 ± 0.0001 pmol/mg (Table 3). The highest VEGF expression was found in the combined occlusal trauma and moderate orthodontic force group with a mean expression of 0.0025 ± 0.0003 pmol/mg (Fig. 4). Kruskal–Wallis test found statistically significant differences among groups (*p* < 0.001) and Mann–Whitney’s U test showed statistically significant differences in all possible pairwise comparisons (*p* < 0.001).

**Table 3:** VEGF expression in human dental pulps of teeth with occlusal trauma, orthodontic force and a combination of both stimuli

Applied Stimuli^b^	No. of teeth	Mean^a^	SD	95% confidence interval	
Healthy premolars control group	10	0.0004	0.0001	0.0003	0.0006
Occlusal Trauma	10	0.0010	0.0001	0.0009	0.0012
Orthodontic Force	10	0.0014	0.0001	0.0013	0.0016
Occlusal Trauma + Orthodontic Force	10	0.0025	0.0003	0.0023	0.0028

^a^Values are presented as VEGF concentration in pmol per mg of dental pulp

The Kruskal–Wallis test showed statistically significant differences among groups (*p* < 0.001)

^b^Mann-Whitney’s U post-hoc pairwise comparisons (with correction of significance) showed statistically significant differences in all possible pairwise comparisons (*p* < 0.001)

## Discussion

SP, CGRP, and VEGF play a crucial role in the defense mechanisms of pulp tissue against any harmful stimuli by activating angiogenesis and neurogenic inflammation that induce reparation through mineralized tissue formation [11, 16, 21].

Control group healthy premolars, that were not subjected to any stimulus, showed SP and CGRP basal levels that allow pulp tissue homeostasis with appropriate oxygen and nutrient supply, which contributes to the proper functioning and maintenance of a healthy pulp. These findings were consistent with the baseline levels in healthy human dental pulps reported in previous studies [18, 19].

VEGF expression in the control group shows the physiological levels in the human dental pulp that are required for tissue response to masticatory function in order to provide oxygen supply for different cell populations based on angiogenic mechanisms [22].

The statistically significant increase in SP, CGRP, and VEGF expressions between the control and experimental groups can be explained similarly, taking into consideration that increased expression of these proteins generates biological defense events that are proportional to the type, nature, and magnitude of the stimulus.

Dental pulp cellular activity increases when subjected to harmful stimuli, thereby producing SP and CGRP [21]. SP binds to the NK1 receptor of endothelial cells, inducing mitotic activity to form new blood vessels. Moreover, SP induces VEGF release, modulating the action of endothelial cells and fibroblasts, allowing cell migration and proliferation, either directly or indirectly, and attracting cells with angiogenic potential [10, 22].

Furthermore, CGRP acts synergistically with SP by increasing inflammatory mediators, compromising the pulp blood flow and collateral circulation due to hypoxia caused by occlusal trauma, orthodontic forces, or a combination of both stimuli, initiating a compensating mechanism to correct the resulting damage, avoiding the vascular collapse that will be higher according to the intensity of the stimulus [19, 23, 24]. CGRP intervenes in the formation of new vessels via the cAMP–PKA pathway activation, which is related to endothelial cell stimulation through its receptor CGRPR1. It also boosts the action of VEGF [25] and fibroblast and odontoblast stimulation to form mineralized tissue as a defense mechanism [4, 26].

SP and CGRP induce VEGF expression to initiate angiogenesis as a defense mechanism of the human dental pulp [7, 9, 23]. This may support other studies’ hypothesis that occlusal trauma and orthodontic forces cause hypoxic areas due to increased cellular activity intended for mineralized tissue formation as a defense mechanism [4].

Angiogenesis is initiated as the VEGF expression level increases, creating vascular destabilization through increased hypoxia-inducible factor-1 [13]. The binding of VEGF to VEGF-R2 is responsible for the differentiation, proliferation, migration, and tubulogenesis of endothelial cells. Subsequently, VEGF bound to VEGF-R1 acts on the stalk cells and is involved in the guidance and control of cell formation in the new vasculature until its stabilization in order to reach normoxia and reduce its expression [12, 14].

Finally, in the occlusal trauma plus the moderate orthodontic forces group, SP, CGRP, and VEGF expressions were statistically significantly higher than those in other groups. It could be inferred from these results that the synergy between the two stimuli affects the pulp microcirculation even more because it compromises blood flow and collateral circulation and creates hypoxia in the pulp tissue, caused by vascular congestion that, if it is not controlled, could lead to necrosis [4, 13, 27, 28].

Therefore, this investigation is relevant because its scope is closer to the patients’ clinical condition under orthodontic treatment, and because patients under this treatment may suffer occlusal interferences during teeth movement [29], this could lead to clinical situations such as altered response to sensitivity tests and reduced lumen and canal size due to dentin formation as a defense mechanism [30, 31]. Neurogenic inflammation and angiogenesis play a crucial role in the defense mechanisms of the human dental pulp against stimuli such as orthodontic forces and occlusal interferences. Evaluation of SP, CGRP and VEGF expression is important to understand and correlate the defense and reparative activity of the pulp.

Due to the in vivo nature of the study, an immediate stimulus model was performed. This prevented the evaluation of SP, CGRP, and VEGF expressions over a more extended period, which is considered as a study limitation. However, neuropeptides and VEGF are released immediately after stimuli, and therefore their effects are also immediate and sustained as well [4]. Moreover, there is evidence on the literature that supports that a 24 h period is enough to provide a stimuli/effect response [19]. Another limitation that could affect the results is the non-compliance of the patients to follow home-instructions. However, patients were carefully informed about following these instructions to improve their compliance.

## Conclusions

Within the limitations of this study, it can be concluded that SP, CGRP, and VEGF expressions significantly increase in human dental pulps when stimulated by occlusal trauma combined with moderate orthodontic forces, as compared with these two stimuli applied independently. The results supports the theory that stimuli are additive on the pulp tissue and therefore a more comprehensive understanding of how neurogenic inflammation and angiogenesis play a role on the defense mechanisms of dental pulp could explain some clinical outcomes when pulps are under stimuli such as the occlusal trauma and orthodontics.

## Data Availability

The datasets used and/or analysed during the current study are available from the corresponding author on reasonable request.

## Abbreviations

SP: Substance P
NK1: Neurokinin 1
CGRP: Calcitonin gene-related peptide
CGRPR1: Calcitonin gene-related peptide receptor 1
CGRPR2: Calcitonin gene-related peptide receptor 2
VEGF: Vascular endothelial growth factor
VEGF-R1: Vascular endothelial growth factor receptor 1
VEGF-R2: Vascular endothelial growth factor receptor 2
